# Response to: ‘Hyperbinding’ in functional movement disorders: role of supplementary motor area efferent signalling

**DOI:** 10.1093/braincomms/fcae465

**Published:** 2025-02-24

**Authors:** Bernhard Pastötter, Anne Weissbach, Adam Takacs, Josephine Moyé, Julius Verrel, Fabian Chwolka, Julia Friedrich, Theresa Paulus, Simone Zittel, Tobias Bäumer, Christian Frings, Christian Beste, Alexander Münchau

**Affiliations:** Department of Cognitive Psychology, University of Trier, 54296 Trier, Germany; Institute for Cognitive and Affective Neuroscience (ICAN), University of Trier, 54296 Trier, Germany; Institute of Systems Motor Science, CBBM, University of Lübeck, 23562 Lübeck, Germany; Cognitive Neurophysiology, Department of Child and Adolescent Psychiatry, Faculty of Medicine, TU Dresden, 01307 Dresden, Germany; Institute of Systems Motor Science, CBBM, University of Lübeck, 23562 Lübeck, Germany; Institute of Systems Motor Science, CBBM, University of Lübeck, 23562 Lübeck, Germany; Institute of Systems Motor Science, CBBM, University of Lübeck, 23562 Lübeck, Germany; Institute of Systems Motor Science, CBBM, University of Lübeck, 23562 Lübeck, Germany; Institute of Systems Motor Science, CBBM, University of Lübeck, 23562 Lübeck, Germany; Department of Neurology, University of Lübeck, 23562 Lübeck, Germany; Department of Neurology, University Medical Center Hamburg-Eppendorf, 20251 Hamburg, Germany; Institute of Systems Motor Science, CBBM, University of Lübeck, 23562 Lübeck, Germany; Department of Cognitive Psychology, University of Trier, 54296 Trier, Germany; Institute for Cognitive and Affective Neuroscience (ICAN), University of Trier, 54296 Trier, Germany; Cognitive Neurophysiology, Department of Child and Adolescent Psychiatry, Faculty of Medicine, TU Dresden, 01307 Dresden, Germany; Institute of Systems Motor Science, CBBM, University of Lübeck, 23562 Lübeck, Germany

We thank Dr Dutta for his thoughtful critique of our paper, ‘Increased beta synchronization underlies perception-action hyperbinding in functional movement disorders’.^1^ The letter raises important points about the role of the supplementary motor area (SMA) in sensory suppression during voluntary movements and its potential relevance to understanding the neural dysfunctions in functional movement disorders (FMD).^2^ Specifically, the SMA is thought to send efferent signals to modulate sensory feedback, that is, the perception of action effects, and reduce sensory input from this feedback during voluntary action.^3^ This mechanism may be particularly relevant to FMD, where patients often struggle to differentiate volitional from non-volitional movements.^4^

In our study, we did not investigate response–effect bindings or sensory suppression. Instead, we focused on stimulus–response (S–R) bindings and demonstrated that beta event-related synchronization over the SMA predicts behavioural S–R binding effects, which were significantly stronger in FMD patients compared to healthy controls. The SMA is increasingly recognized as a key brain region for integrating perception and action.^5^ This applies to both the integration of externally perceived stimuli with responses (as in S–R bindings) and the integration of responses with perceived effects or sensory effect anticipation (as in response–effect bindings). In fact, these dual functions align with ideomotor principles, where the anticipation of effects drives motor behaviour.^6^ Thereby, it has been suggested that SMA (and pre-SMA) activations are directly linked to the complexity of perception–action associations.^5^

Following this suggestion, increased SMA beta synchronization may reflect a more complex perception–action association network in FMD patients compared to healthy controls, which may in turn arise from the fact that FMD patients not only bind relevant stimulus information to responses but also irrelevant stimuli or stimulus features. In our study, for example, task-irrelevant features such as the colour and position of stimuli (note that only the orientation of stimuli was task relevant) may have been more likely to be bound into S–R associations in FMD patients than in healthy controls. This propensity to bind irrelevant features likely contributes to difficulties in shielding intentions from competing goals, potentially leading to disorganized or inefficient responses.^7^

Thus, a significant open question is whether patients with FMD exhibit a general tendency towards ‘hyperbinding’ or if this tendency is particularly pronounced when irrelevant stimuli are involved. Future studies, especially those employing tasks like the distractor–response binding task^8,9^ should explore whether the inclination to integrate irrelevant stimuli plays a key role in the disorder—both behaviourally and in electrophysiological data, particularly regarding post-movement beta event-related synchronization.

Dr Dutta’s point about reduced sensory suppression fits well with the complexity argument regarding the processing of S–R and response–effect bindings in the SMA. Less effective sensory suppression could contribute to increased complexity in perception–action association networks, as irrelevant stimuli and features become more likely to influence response selection. We agree that this interplay warrants further investigation to advance the pathophysiological understanding of FMD and other movement disorders, such as Parkinson’s disease.^10^

## Data Availability

Data sharing is not applicable to this article as no new data were created or analysed.

## References

[1] Pastötter B, Weissbach A, Takacs A, et al Increased beta synchronization underlies perception-action hyperbinding in functional movement disorders. Brain Commun. 2024;6(5):fcae301. 39386091 10.1093/braincomms/fcae301 PMC11462440

[2] Dutta A . ‘Hyperbinding’ in functional movement disorders: role of supplementary motor area efferent signalling. Brain Commun. In Press. 10.1093/braincomms/fcae464 PMC11848267 39995655

[3] Haggard P, Whitford B. Supplementary motor area provides an efferent signal for sensory suppression. Brain Res. 2004;19(1):52–58. 10.1016/j.cogbrainres.2003.10.018 14972358

[4] Edwards MJ, Moretto G, Schwingenschuh P, Katschnig P, Bhatia KP, Haggard P. Abnormal sense of intention preceding voluntary movement in patients with psychogenic tremor. Neuropsychologia. 2011;49(9):2791–2793. 21683724 10.1016/j.neuropsychologia.2011.05.021

[5] Nachev P, Kennard C, Husain M. Functional role of the supplementary and pre-supplementary motor areas. Nat Rev Neurosci. 2008;9(11):856–869. 18843271 10.1038/nrn2478

[6] Hommel B . Ideomotor action control: On the perceptual grounding of voluntary actions and agents. In: Prinz W, Beisert M, Herwig A, eds. Action science: Foundations of an emerging discipline. The MIT Press; 2013:113–136.

[7] Huys ACML, Bhatia KP, Edwards MJ, Haggard P. The flip side of distractibility—Executive dysfunction in functional movement disorders. Front Neurol. 2020;11:969. 33041967 10.3389/fneur.2020.00969 PMC7517871

[8] Frings C, Rothermund K, Wentura D. Distractor repetitions retrieve previous responses to targets. Q J Exp Psychol. 2007;60(10):1367–1377. 10.1080/17470210600955645 17853245

[9] Pastötter B, Moeller B, Frings C. Watching the brain as it (un)binds: Beta synchronization relates to distractor-response binding. J Cogn Neurosci. 2021;33(8):1581–1594. 34496371 10.1162/jocn_a_01730

[10] Meppelink AM, de Jong BM, Beudel M. Internal and external modulation of parieto-premotor circuitry in movement disorders. Brain Commun. 2024;6(5):fcae339. 39386088 10.1093/braincomms/fcae339 PMC11462435

